# Epidemiology of CoViD-19 Pandemic: Recovery and mortality ratio around the globe

**DOI:** 10.12669/pjms.36.COVID19-S4.2660

**Published:** 2020-05

**Authors:** Aziz Ullah Noor, Farhana Maqbool, Zulfiqar A. Bhatti, Asmat Ullah Khan

**Affiliations:** 1 Aziz Ullah Noor, Department of Microbiology, Faculty of Health Sciences, Hazara University, Mansehra, Khyber Pakhtunkhwa, Pakistan; 2 Farhana Maqbool, Ph.D. Department of Microbiology, Faculty of Health Sciences, Hazara University, Mansehra, Khyber Pakhtunkhwa, Pakistan; 3 Zulfiqar A. Bhatti, Ph.D. Department of Environmental Science, COMSATS University, Abbottabad Campus, 22060 Pakistan; 4 Asmat Ullah Khan, Department of Human Anatomy, Southern Medical University, Guangzhou, P. R. China

**Keywords:** CoViD-19, Pandemic, Public health, Wuhan, Zoonotic

## Abstract

Coronavirus Disease 2019 (CoViD-19) is the third type of coronavirus disease after severe acute respiratory syndrome (SARS) and Middle East respiratory syndrome (MERS) that appears in human population from the past two decades. It is highly contagious and rapidly spread in the human population and compelled global public health institutions on high alert. Due to genetic similarity of this novel coronavirus 2019 with bat virus its emergence from bat to humans is possible. The virus survive in the droplets of coughing and sneezing and spread around the large areas through infected person resulting in its rapid spread among people. Clinical symptoms of CoViD-19 include fever, dry cough, dyspnea, loose stool, nausea and vomiting. The present review discuss the origin of CoViD-19, its rapid spread, mortality rate and recoveries ratio around the world. Since its origin from Wuhan, the CoViD-19 spread very rapidly all across the countries, on April 17, 2020 this disease has affected 210 countries of the globe. The data obtained showed over 2.4 million confirmed cases of CoViD-19. Higher mortality rate was found in Algeria and Belgium as 15% and 13.95%, respectively. Lower mortality rate was found in Qatar 0.17% and Singapore 0.2%. Recovery versus deceased ratio showed that recovery was 68, 59 and 35 times higher than the death in Singapore, Qatar and Thailand respectively. It is concluded that 2019-novel corona virus is a zoonotic pathogen similar to MERS and SARS. Therefore, a barrier should be maintained between and across the human, household and wild animals to avoid such pandemics.

## INTRODUCTION

Coronaviruses (CoVs) are extremely important, widely distributed pathogens found in both humans and mammals. These are enveloped, single-stranded RNA viruses belonging to family *Coronaviridae*, circulating in birds and mammals and affecting human, domestic and wild animals.^1^ Two coronaviruses related human diseases emerged previously in 2003 and 2012, have been named as SARS and MERS respectively. These two strains of corona viruses collectively affected 10,000 people with a fatality rate of 10% for SARS-CoV and 37% for MERS-CoV.^2^,^3^

During the last 50 years, the emergence and re-emergence of deadly infectious diseases have increased. SARS-CoV-1 disease was originated in Guangzhou city of China and the start of 2020 was again a challenging year for this country because of extremely contagion 2019-novel coronavirus (2019-nCoV) disease outbreak.^4^ This virus is also known as SARS-CoV-2 because of its same place of origin and genetic similarity with slight mutation in SARS-CoV-1 strain.

In December, 2019 a series of new cases of pneumonia were reported in Wuhan, China, whose clinical presentation were resembled to viral pneumonia.^5^ Deep sequencing helped to diagnose the new virus, named as 2019 novel coronavirus (2019-nCoV) by World Health Organization (WHO). The origin of this virus has been reported in Wuhan, China. Few recent reports suggested its transmission from animal to human and within humans, which signifies its zoonotic potential.^6^ So far, over 2.4 million cases have been reported worldwide with a total fatality reached to over two lac till April 26, 2020. The virus has been reported from many other countries mainly due to the traveling of infected/suspected people from China to these countries. Chinese health ministry took immediate action to investigate and control the disease, including quarantine measures, continuous observation of contacts, clinical and epidemiological data collection from infected people and development of diagnostic tools and efficient treatment protocols. Beside these measures Chinese government had recommended to immigrant not to travel back to home country and should stay in 14 days quarantine before leaving to stop its further spread but many countries have not paid attention on this advice as a result coronavirus spread globally. This review focuses on origin of 2019-nCoV, incidence of CoViD-19 in China, its clinical manifestations, mortality and recovery around the globe.

### Origin of 2019-novel corona virus

The source of origin of coronavirus is still a mystery, however, early investigations have reported its possible origin from the Wuhan Seafood Wholesale Market. Most of the early patient history associated with their movement to that Seafood Market. Whereas, there were numerous other patients who have not gone to that market in those days. That association indicated its human to human transmission in spreading the outbreak. Few environmental samples taken from the market have been reported positive but no specific animal was identified as its origin.^7^ An initial investigation based on codon claimed snake as an origin.^8^ It has also been proposed, that 2019-nCoV naturally propagates in bats.^9^ Previous study revealed that wet markets of southern China including Wuhan and Guangzhou cities have the greater risk of spreading novel corona viruses, because of wild animal trading and the absence of biosecurity measures.^10^ The other possibility is that bats and their excrements are commonly used in Traditional Chinese Medicine, which may also be a source of infection.^11^ It is also possible that the virus had infected other mammal that was traded at the market and served as the source of the infection to people.

**Fig.1:**
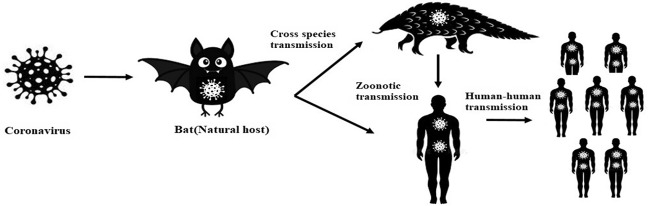
Transmission flow chart of CoViD-19.

Two already highly pathogenic coronaviruses were reported to be originated from the animals. The transmission of the first highly pathogenic virus, SARS-CoV occurred from animal to human in Wet markets. The source of SARS-CoV was bat which transferred this virus to human via civet cat as an intermediate host.^12^ The bats were also reported to be the possible origin of MERS-CoV, which is also a zoonotic virus.^13^ However, MERS-CoV was reported in the patients, having frequent contact with the camels in the Middle East. Dromedary Camel was considered as traditional household animal, to avoid contact with camel is not possible due to which they suffered from the periodic outbreak of MERS-CoV.^14^ In this context, the identification of source animals, responsible for the transmission of CoViD-19 virus is extremely essential in order to control and prevent any future outbreak. The possible transmission cycle of CoViD-19 is presented in Fig.1.

### Current Outbreak of CoViD-19 in China

CoViD-19 is a great public health and safety concern all across the globe and the Chinese government, international agencies and WHO is aware of the consequences of the outbreak. The first case of CoViD-19 outside China was reported in Thailand.^15^

Due to rise in infected and suspected cases of CoViD-19 in China by the end of the third week of January 2020, the Chinese government had completely lockdown the Wuhan city, Hubei province. They partially lockdown other cities at higher risk to stop its further spreading. Since the public health emergency declared by WHO, much more attention at national and international levels has been focused on CoViD-19. The concept of One Health which means that ‘the health of people is linked with the animal and connected environment’ has gained great importance in this current scenario.^16^,^17^

The super-spreaders (infected person who travels and infect others) are mostly responsible for large outbreaks. For example, traveling of one infected person with SARS-CoV from Hong Kong to Toronto infected 128 people in a local health care facility where he visited for treatment. The same situation was with MERS-CoV, where a single patient from Saudi Arabia traveled to South Korea infected 186 patients with MERS-CoV.^18^

The epidemiological characteristics observed in the case of CoViD-19 varied to a great extent as compared to the SARS-CoV. The 2019-nCoV replicates efficiently in the upper respiratory tract, but the onset of symptoms appears to be slow. Although the infected individual carries a huge number of virus still he/she can perform his/her normal activities, which leads to the spread of this infection to other individuals. Whereas, in the SARS-CoV patients, the transmission of infection is not reported during the prodromal period and transmission occurs when the patient becomes severely ill.^7^

Various factors affect and determine the spread of disease in an outbreak including transmissibility and severity. Morbidity and mortality will rely on the combination of transmissibility and severity and a rapid person to person transmission occurred in this outbreak. The epidemiologists estimated the R_O_ (the basic reproduction number to measure the transmissibility) value would be 2.2 for CoViD-19.^19^

The severity and the case-fatality rate could be underestimated because many infected people have not yet recovered and may deceased.

### Vertical transmission of CoViD-19

After the onset of the CoViD-19 outbreak, the potential risk of vertical transmission was a question mark. Control and prevention of this novel emerging infection among pregnant women have become a primary concern regarding vertical transmissibility. The latest study published in The Lancet gives some insight into the clinical features during pregnancy and potential of vertical transmission CoViD-19 infection in pregnant women.^20^ Although the research manipulates only a small number of nine pregnant women with confirmed CoViD-2019, no evidence for intrauterine infection was reported in late pregnancy in these cases.^21^

A SARS-CoV-2 genomic study suggested that this virus has 88% genetic similarity with two SARS-like coronaviruses isolated from a bat, Sbat-SL-CoVZC45 and bat- SL-CoVZXC21. While in case of SARS-CoV-1 and MERS-CoV bat’s genome similarities were 79% and 50% respectively. Homology studies have shown that SARS-CoV-2 has a similar receptor binding structure to that of SARS-CoV-1. So, the ability of vertical transmission of corona-19 could be as low as that of SARS-CoV-1.^22^

Although 2019-nCoV has adverse effects on newborn including shortening of breathing, premature birth and liver disorder but its vertical transmission has not proved.^23^ The clinical findings of CoViD-19 infected pregnant women were same as non-pregnant women infected with CoViD-19 infection.^24^

### Clinical manifestations

In accordance with SARS-CoV and MERS CoV, this new virus was observed to affect more males than female individuals. The reduced susceptibility of women can be explained by the fact that the sex hormone and X chromosome play an important role in innate and adaptive immunity. Moreover, almost half of the population infected by CoViD-19 has underlying problems mainly diabetes, cerebrovascular and cardiovascular problems similar to MERS-CoV. The older people with the weak or compromised immune system are more susceptible to this disease. 24

Among the clinical signs; fever, dry cough, dyspnea, and fatigue were common in all cases. Upper respiratory tract infection i.e. rhinorrhea, sneezing, or sore throat were also uncommon in CoViD-19. The laboratory reports of CoViD-19 patients indicates lymphopenia (decrease in white blood cells) which suggested the destruction of lymphocyte and other immune cells by coronavirus leading to the weakening of cellular immune system.^25^ Some patients suffer from acute respiratory distress and septic shock which lead to the failure of multiple organs.^24^-^26^ These cases are of crucial importance to be treated at earlier stages. CT scan indicated ground-glass opacification and occasional consolidation in the patients.^27^

Most of the deaths have been reported in the patients who have the characteristic of warning signs described by the Multi Logistic Binary Search Tree analysis (MuLBSTA) model. These six signs which are included in the MuLBSTA model are multinodular infiltration, lymphopenia, bacterial co-infection, smoking history, hypertension and age.^28^

### Treatment and prevention

Few or no options are available for treating a viral disease that emerged suddenly, no vaccine has been developed yet for the prevention of CoViD-19 infection.^26^ A combination of antiviral drugs Lopinavir/Ritonavir, PEGylated interferon, and ribavirin has been used in a MERS-CoV case reported in South Korea, that helped in the successful clearance of the virus.^29^ Another viral drug, remdesivir has been reported to be effective against the viral infection. *In-vitro* studies indicated that Remdesivir has been successful in the termination of viral RNA replication,^30^,^32^ and showed effectiveness against the MERS-CoV, SARS-CoV and other bat originated coronaviruses.^31^,^33^

Qamar et al., 2020 screened the database of 32,297 Chinese medicinal plants for their antiviral activity. They suggested 9 medicinal plants that might help in the prevention of viral replication.^34^ Further studies are necessary to figure out the effectiveness of these plants in this infection. Another study on virtual screening of a database of more than 3000 Food and Drug Administration (FDA) approved drugs was carried out in order to find the possible best available drug. The results suggested that protein inhibitors in Human Immunodeficiency Virus (HIV) drugs might be helpful against the CoViD-19.^35^ Recently FDA have authorized the use of hydroxychloroquine and chloroquine due to emergency situation without double blind and clinical trial for the treatment of CoViD-19.^36^

### Mortality and recovery ratio around the globe

Since the discovery of the virus, the CoViD-19 spread very rapidly all across the countries and cases have been reported in 210 countries around the globe (till 10:39 GMT on April 26, 2020). The data obtained showed over 2.4 million confirmed cases of CoViD-19.^37^ Higher mortality rate (15%) was found in Algeria, Belgium (13.95), Italy and United Kingdom (13%) and Netherland (11.35%). Lower mortality rate was found in countries Qatar 0.17%, Singapore 0.2%, United Arab Emirate 0.6%, and Australia 0.97 . The WHO keeps on updating and sharing these figures on daily basis and till April 28^th^ it had iussued ninety seven reports giving countrywise details of number of cases.

Higher mortality rate is related with the total number of infected cases, as significant positive correlation r=0.9, n=56 was found between confirmed cases and deaths, which showed that disease spread increases the risk of death due to overcrowded hospitals, lower availability of medical facility and other environmental factors. Before mitigation measures were taken place CoViD-19 was already spread in the early stages.^38^ Countries showed early response suffered less than the countries that did not care in the early stage of this pandemic. Yet another reason of this pandemic was as 80% of CoViD-19 cases are mild or asymptomatic so the symptom base control of this disease is very difficult and less effective.

Recovery versus deceased ratio was calculated and the data showed that recovery was 68, 59 and 35 times higher than the death in Singapore, Qatar and Thailand respectively. Lower value of deceased over recovery ratio was found in United Kingdom (0.03), Netherland (0.08), Ireland (0.16) and Norway (0.21).^37^ In contrast to CoViD-19 prevalence, previous study shows that community acquired pneumonia cases were high in male who belonged to lower socio-economic group, illiterate people living in rural areas.^39^ Patients recoveries are significantly correlated with the number of cases (r = 0.63, n = 56), showed that recoveries are increasing with increase in number of cases. The potential factors involved in the recovery might be strong immune system among the population, good dietry habits and early treatment and Bacillus Calmette-Guérin (BCG) vaccination policy in some country showed less cases than without BCG vaccinated nations. 40

According to Reuter the Global death due to COVID19 disease passed two hundred thousand on April 25^th^ 2020 and it is expected that the total number of infected cases will cross three million people by the end of April 2020. More than 50% of these deaths have occurred in United States, Spain and Italy the worst hit countries. Till April 25^th^ United States had reported fifty two thousand four hundred deaths while Italy, Spain and France have reported between 22,000 and 26,000 fatalities each. Of the top twenty most severely affected countires Belgium has reported highest number of fatalities per capita with six deaths per ten thousand people compared to 4.9 in Spain and 1.6 in the United States. The overall mortality rate of cases reported in United States has been 8% while more than 10% cases reported in Spain and Italy have resulted in Deaths. Asia and Latin America have each reported more than seven thousand deaths while Middle East has reported more than eight thousand eight hundred deaths.^41^

## CONCLUSION

The danger of CoViD-19 can be argued by the fact that this virus has inherited the ability to mutate. It is accepted that most viruses survived in their natural reservoir for a longer time. Therefore, the most fruitful method to prevent viral zoonosis is to preserve the barriers between human society and natural reservoir of viruses. Global preparedness for any outbreak has been suggested that national health security plan is alarmingly weak around the world and we should prepare ourselves better for the future.

### Author`s Contribution

**AUN:** Collection of basic information.

**FM:** Review writing and editing.

**ZAB:** Updated data collection and organization, helped in writing.

**AUK:** Helped in writing and performed statistical analysis.
